# Fabrication of a Disposable Amperometric Sensor for the Determination of Nitrite in Food

**DOI:** 10.3390/mi14030687

**Published:** 2023-03-20

**Authors:** Chao Liu, Daoming Chen, Chunnan Zhu, Xiaojun Liu, Yu Wang, Yuepeng Lu, Dongyun Zheng, Baorong Fu

**Affiliations:** 1 College of Biomedical Engineering, South-Central Minzu University, Wuhan 430074, China; 2 Hubei Key Laboratory of Medical Information Analysis & Tumor Diagnosis and Treatment, Wuhan 430074, China; 3 Key Laboratory of Cognitive Science, State Ethnic Affairs Commission, Wuhan 430074, China; 4 Wuhan Institute for Food and Cosmetic Control, Wuhan 430040, China; 5 Key Laboratory of Edible Oil Quality and Safety for State Market Regulation, Wuhan 430040, China; 6 Wuhan Great Sea Hi-Tech Co., Ltd., Wuhan 430223, China

**Keywords:** silver nanoparticles, green synthesis, tea polyphenols, pencil graphite electrode, nitrite

## Abstract

Silver nanoparticles (AgNPs) were synthesized through an environmentally friendly method with tea extract as a reduction agent. By immobilizing them on the surface of a low-cost pencil graphite electrode (PGE) with the aid of a simple and well-controlled in-situ electropolymerization method, a novel nanosensing interface for nitrite was constructed. The film-modified PGE showed good electrocatalytic effects on the oxidation of nitrite and was characterized through scanning electron microscopy, X-ray photoelectron spectroscopy, and electrochemical techniques. Characterization results clearly show that the successful modification of AgNPs improved the surface area and conductivity of PGEs, which is beneficial to the high sensitivity and short response time of the nitrite sensor. Under the optimal detection conditions, the oxidation peak current of nitrite had a good linear relationship with its concentration in the range of 0.02–1160 μmol/L with a detection limit of 4 nmol/L and a response time of 2 s. Moreover, the sensor had high sensitivity, a wide linear range, a good anti-interference capability, and stability and reproducibility. Additionally, it can be used for the determination of nitrite in food.

## 1. Introduction

Nitrite is a common chromogenic agent and preservative in meat food because it can not only make the color of meat look better, but also inhibit the generation of botulinum toxin [1]. However, the excessive intake of nitrite will lead to methemoglobinemia [2] and gastric cancer [3]. Therefore, monitoring whether the amount of nitrite in food is excessive is essential to protect human health, requiring the accurate and timely monitoring of nitrite in food samples. Some methods for the determination of nitrite have been proposed in recent years, such as spectrophotometry [4], the chemiluminescence method [5], capillary ion chromatography [6], capillary electrophoresis [7], the fluorescence method [8], the electrochemical sensing method [9], etc. Particularly, the electrochemical sensing method has attracted more attention because of its simple operation, high sensitivity, and rapid response time.

Through previous researchers’ efforts, a number of well-performing electrochemical sensors have been developed for the determination of nitrite. For example, Ren et al. [10] proposed an electrochemical nitrite sensor based on MoS_2_ nanosheets/carboxylic multiwall carbon nanotube- (MoS_2_/MWCNT-COOH) modified glassy carbon electrodes. The sensor has high sensitivity, good reproducibility, and good stability and can be used for the accurate detection of nitrite in water samples. Zhang et al. [11] prepared a graphene oxide-gold nanostar-modified glassy carbon electrode and used it for the electrochemical sensing of nitrite. With good stability and reproducibility, the sensor was applied to the detection of nitrite in water samples with good selectivity. The performances of the reported electrochemical nitrite sensors were satisfactory, however, it would be better if the manufacturing process were simplified and the cost reduced. Therefore, to obtain a higher convenience and lower cost, developing disposable nitrite sensors is important.

A good alternative can be found in pencil graphite electrodes (PGEs), which have been presented as the cheapest and most commercially available electrodes [12]. The uniqueness of PGEs is attributed to some of their attractive properties, such as mechanical rigidity, ease of modification, miniaturization, and rapid surface renewal compared to other commonly used electrodes that involve complicated surface-polishing procedures [13]. Recently, PGEs have been used advantageously in a variety of detection processes, including the detection of free residual chlorine [14], SARS-CoV-2 [15], and 4-nitrophenol [16]. In 2020, Mejri et al. developed a well-performing nitrite detection platform based on a poly(curcumin)/gold foam/molybdenum disulfide nanosheet-modified PGE [17]. However, the construction process is complicated. Therefore, developing novel nitrite-sensitive materials and immobilizing them through a simple operation on the surface of PGEs is essential for the fabrication of disposable nitrite sensors.

Due to its perfect conductivity, large specific surface area, and good electrocatalytic activity, silver nanoparticles (AgNPs) are considered an ideal sensing material and have been widely used in constructing sensors for the detection of glucose [18], furaneol [19], and H_2_O_2_ [20]. Some electrochemical nitrite sensors using AgNPs as a sensitive material have been reported [21,22,23]. However, all the AgNPs in above reports were synthesized through chemical methods that are not environmentally friendly and will lead to poor biocompatibility among AgNPs. Some green synthesis methods for fabricating AgNPs have been proposed and are well studied, but their applications in the electrochemical sensing area are seldom reported.

In this work, AgNPs were synthesized by reducing AgNO_3_ with tea extract as a reduction agent instead of sodium borohydride or potassium borohydride. This method is attractive because of its no-pollution, low-cost, and simple operation. The obtained AgNPs were modified on the surface of a PGE through co-electropolymerization with tea polyphenols. Results from electrochemical techniques indicate that the modified PGE shows positive electrocatalytic effects on the oxidation of nitrite and could be used as a disposable nitrite sensor. Compared with other reported electrochemical nitrite sensors, this sensor boasts a higher performance and can be applied for the accurate and timely detection of nitrite in food samples.

## 2. Materials and Methods

### 2.1. Chemicals and Apparatus

Absolute ethanol, AgNO_3_, NaNO_2_, NaNO_3_, HNO_3_, NaOH, H_3_PO_4_, NaH_2_PO_4_, Na_2_HPO_4_, MgCl_2_, CaCl_2_, K_2_ (Fe(CN)_6_), and K_3_ (Fe(CN)_6_) were purchased from the Sinopharm Chemical Reagent Co., Ltd. (Tianjin, China). All reagents were of analytical grade and used without further purification. All solutions were prepared with ultra-pure water. A AgNO_3_ standard solution with a concentration of 0.1 mol/L (M) was prepared and stored in a refrigerator away from light. Carbon paper was purchased from the Gaoshiruilian Photoelectric Technology Co., Ltd. (Tianjin, China) Touch-line 0.7 mm diameter 2B pencil leads, copper wire, silver epoxy glue, and epoxy glue for making electrodes were purchased from a local market (Wuhan, China). Enshi selenium tea was purchased from local supermarket. Disposable needle suction filters and microporous membranes (Φ = 0.22 μm) were purchased from the Xinshenshi Chemical Technology Co., Ltd. (Wuhan, China).

Cyclic voltammetry, differential pulse voltammetry, and amperometric I-t curve methods were used for electrochemical experiments and carried out with an electrochemical workstation (CHI660D, Chenhua Instrument Co., Ltd., Shanghai, China). The pH value of the supporting electrolyte was adjusted with an acidity meter (pHS−3E, Youke Instrument Co., Ltd., Shanghai, China). Electrochemical impedance spectroscopy characterization was carried out at an IM6ex electrochemical workstation (Zahner, Kronach, Germany). Scanning electron microscopy (SEM) was performed using the SU8010 field emission scanning electron microscope. The optical properties of AgNPs were investigated on a UV–Vis spectrophotometer (UV−3600i Plus, Shimadzu Corporation, Tokyo, Japan). Transmission electron microscopy (TEM) characterization was performed using the Talos F200X scanning transmission electron microscope (Thermo Fisher Scientific, Waltham, MA, USA). X-ray photoelectron spectroscopy (XPS) characterization was performed using an X-ray photoelectron spectrometer (EscalabXi+, Thermo Fisher Scientific, Waltham, MA, USA).

### 2.2. Green Synthesis of AgNPs Using Tea Extract

Tea extract was prepared according to Qing’s report [24]. Briefly, 5 g Enshi selenium tea leaves were immersed into 50 mL of ultrapure water and heated at 60 °C for 5 min. Then, the mixture was filtered and the filtrate was centrifuged at 10,000 rpm for 5 min. Through purifying the supernatant using suction filtration with a microporous membrane (Φ = 0.22 µm), the tea extract was obtained and stored in a refrigerator under 4 °C for further use within one week.

**Figure 1:**
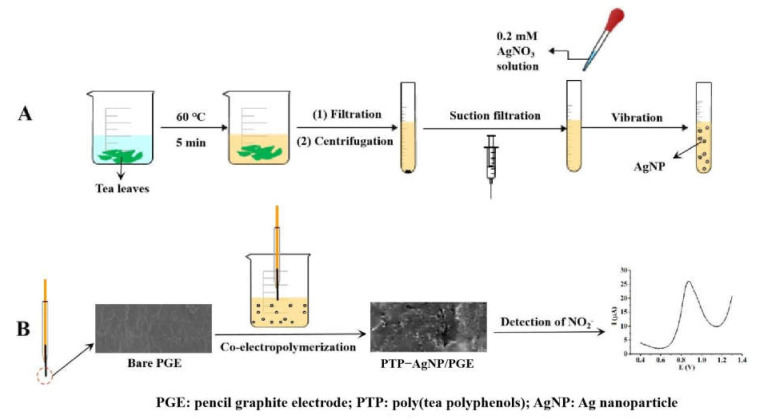
Diagram of the green synthesis processes of AgNPs with tea extract (**A**) and the fabrication procedures of PTP−AgNP/PGE (**B**).

For the green synthesis of AgNPs, an amount of 0.2 mM AgNO_3_ was added into 5 mL of tea extract. After vibrating the solution for 5 min at room temperature, its color rapidly turned from yellow to brown, showing the generation of AgNPs. In the green process for the preparation of AgNPs, tea extract was used as both a reducing agent and a stabilizing agent. Tea extract contains a large number of tea polyphenols with antioxidant functions, which offers the possibility of a direct reduction in silver ion [25]. The presence of other organic compounds, including caffeine, polysaccharide, and tannic acid, in the tea extract allows for the stabilizing of emerging suspensions without adding other reagents that inhibit the growth of silver agglomerates [26]. A diagram for the preparation of tea extract and its application in the green synthesis of AgNPs is shown in Figure 1A.

### 2.3. Preparation of Different Electrodes

The nitrite sensor was prepared as follows: a bare PGE with a surface area of 11.38 mm^2^ was prepared according to the previous report [27]. Briefly, pieces of pencil lead (Φ = 0.7 mm) with lengths of 2.3 cm were adhered to copper wires with silver epoxy glue and air-dried for about 24 h. The pencil lead–copper wire joint was inserted into a plastic pipette tip with 5 cm of the copper wire exposed outside, and then sealed with insulated epoxy glues. The exposed pieces of pencil lead were cut to a length of 5 mm before use. Then, through sweeping it in the tea extract solution containing AgNPs from −0.8 V to 1.8 V at a scan rate of 50 mV/s for 20 cycles, the poly- (tea polyphenols—PTPs) and-AgNP nanocomposite film was immobilized on its surface. The fabricated PTP–AgNP/PGE can be used as a nitrite electrochemical sensor. A diagram for the fabrication of the sensor is shown in Figure 1B.

For further comparison, PTP/PGE was also fabricated following similar procedures as described above except by using a tea extract solution without AgNPs.

To carry out XPS characterization, PTP–AgNP-modified carbon paper was prepared through a similar operation, except that the bare PGE was replaced with carbon paper.

In recent years, a lot of functional monomers with phenol units in their structures have been used to fabricate conductive polymers. Due to their unique electronic and optical properties together with their environmental stability and mechanical durability, conductive polymers have attracted considerable attention [28]. For fabricating conductive polymer-modified electrodes, electropolymerization techniques are a wise choice. Conductive polymers constructed through the electropolymerization method show excellent mechanical, electrical, and chemical properties, which are very meaningful for developing versatile and efficient sensing materials [29]. In addition, the fabricated polymers are uniform and adhere strongly to electrode surfaces. Particularly, in the process of electropolymerization, direct control of the film’s thickness along with uniformity of the material is attainable [30]. The conductive polymer-modified electrodes produce perfect electrocatalytic effects and have been widely applied toward the construction of electrochemical sensors and biosensors [31,32,33,34,35].

There are many phenol units in the structure of tea polyphenols. The above conclusion provides guidance for the electropolymerization of tea polyphenols on the surface of electrodes and its possible applications for the field of electrochemical sensing. The electropolymerization mechanism of phenol red on the surface of glassy carbon electrode has been discussed in detail in Hsieh’s report [36]. An investigation into the polymerization process of phenol red suggests that the radical cations generated from the scans in anodic direction first coupled to a dimer and then propagated further into the resultant polymer. The formation of PTP on PGEs through the electropolymerization method performed in this work may have a similar mechanism.

### 2.4. Electrochemical Measurements

All electrochemical measurements were performed on a CHI 660D electrochemical analyzer (Shanghai Chenhua Co., Ltd., Shanghai, China), which was arranged in a conventional three-electrode pattern and equipped with a platinum wire counter electrode, a saturated calomel electrode (SCE), a reference electrode, and a modified PGE working electrode. The entire electrolytic cell device was placed in a Faraday shielding box with a microcurrent amplifier (Shanghai Chenhua Co., Ltd., Shanghai, China) to avoid external electromagnetic interference. An amount of 0.1 M phosphate buffer (pH = 4.0) was used as the supporting electrolyte solution. The applied potential range was from 0.2 V to 1.3 V.

## 3. Results and Discussion

### 3.1. Characterization of AgNPs

**Figure 2:**
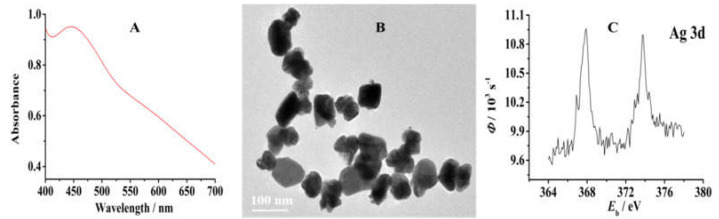
UV–Vis absorbance spectrum (**A**), TEM images (**B**), and XPS spectrum (**C**) of AgNPs synthesized via tea extract.

The maximum absorbance wavelength of 444 nm [37] in the UV–Vis spectrum shown in Figure 2A indicates the successful formation of AgNPs in tea extract. The transmission electron microscopy characterization shows that the green-synthesized AgNPs are bar-shaped with an average particle size of 80 nm, which is shown in Figure 2B.

XPS was used to carry out the chemical analysis of a co-electropolymerization film of PTP–AgNP on carbon paper. Figure 2C shows that the Ag 3d spectrum resolved into two spin-orbit components. The Ag 3d_5/2_ and 3d_3/2_ peaks occurred at BEs of 367.4 and 373.4 V, respectively, corresponding to metallic silver [38], which indicates that AgNPs can be modified successfully on the surface of a base electrode through co-electropolymerization with tea polyphenols.

### 3.2. Morphological Characterization of Different Electrodes

**Figure 3:**
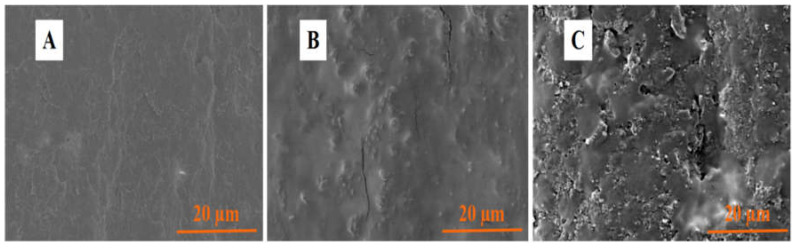
SEM images of bare PGE (**A**), PTP/PGE (**B**) and PTP–AgNP/PGE (**C**).

The surface morphologies of different electrodes were characterized by FE-SEM (Figure 3). As shown in Figure 3A, a typical graphite-sheet structure occurs on the surface of a bare PGE. An obvious film was observed on the surface of PTP/PGE; however, the film is compact (Figure 3B). The co-electropolymerization of AgNPs and PTP gives the electrode surface a loose and porous structure (Figure 3C), which is beneficial for increasing the electrode-effective area and for the enrichment of analyte. The obvious morphological differences in the PTP–AgNP/PGE compared to the others also provides evidence for the successful co-electropolymerization of PTP and AgNPs, which is consistent with the results from the XPS characterization.

### 3.3. Electrochemical Impedance Spectroscopy Characterization of Different Electrodes

**Figure 4:**
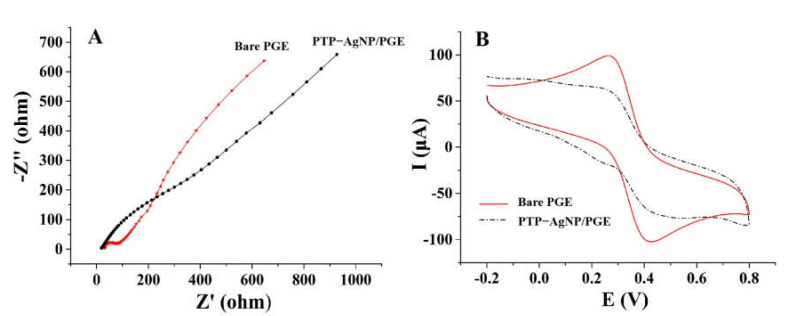
Electrochemical impedance spectroscopy (**A**) and cyclic voltammograms (**B**) of different electrodes in 5 mM Fe (CN)_6_^3−/4−^ and 1 M KCl solution.

Electrochemical impedance spectroscopy (EIS) is an important multifrequency AC electrochemical-measurement technique based on interfacial reactions on the electrode surface. It measures the electrical resistance of the electrode–solution interface over a wide range of frequencies (from 1 mHz to 10 kHz). Here, by taking K_2_ (Fe (CN)_6_) and K_3_ (Fe (CN)_6_) as the probe, EIS was used to evaluate the electrical impedance of different electrode surfaces; the results are shown in Figure 4A. The large radius of PTP–AgNP/PGE in the low-frequency region indicates its higher polarization resistance, which may be due to the low conductivity of PTPs and the electrostatic repulsion between the PTP and the probe. These results are consistent with those of cyclic voltammetry (Figure 4B) and those in Hsieh’s report [36].

### 3.4. Determination of Nitrite with Different Electrodes

#### 3.4.1. Electrochemical Responses of Nitrite on Different Electrodes

**Figure 5:**
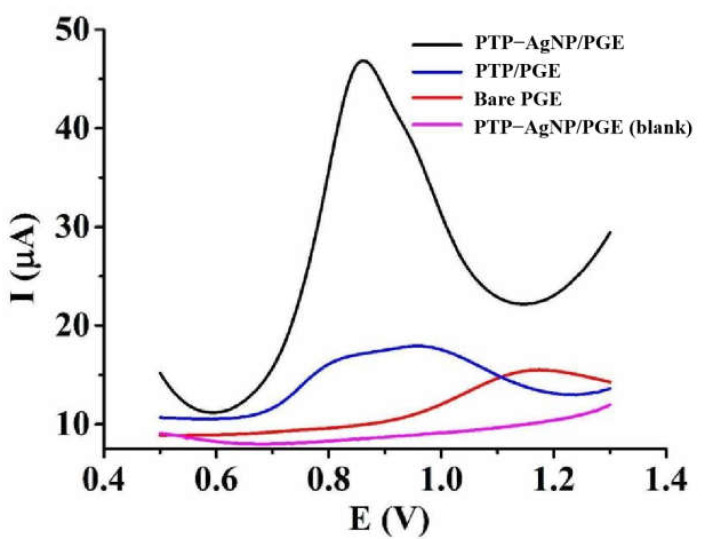
Differential pulse voltammograms between 0 mM and 2 mM of nitrite on different electrodes in 0.1 M PB (pH = 4.0).

With the aid of differential pulse voltammetry, the electrocatalytic effects of PTP–AgNP nanocomposite film on the oxidation of NO_2_^−^ were identified. Figure 5 shows the results of the experiment. When there was no NO_2_^−^ in the supporting electrolyte, no electrochemical response was observed. Using a bare PGE to detect 2 mM NO_2_^−^, only a weak peak appeared with an oxidation peak potential (E_p_) of 1.14 V and an oxidation peak current (I_p_) of 2.21 μA. On the surface of PTP/PGE, the electrochemical response of 2 mM was improved: E_p_ = 0.95 V, I_p_ = 6.04 μA. However, the shape of the peak was very wide. In comparison, a better electrochemical signal of 2 mM was observed on the PTP–AgNP/PGE—E_p_ = 0.86 V, I_p_ = 29.8 μA—indicating good electrocatalytic effects from PTP−AgNP nanocomposite film. Owing to their small size and good conductivity, AgNPs can not only provide a larger surface area for the enrichment of NO_2_^−^, but also accelerate the electron transfer rate between NO_2_^−^ and the sensor. Moreover, PTP is an oligomer that can decrease proton-giving abilities and increase electrocatalytic abilities. In the supporting electrolyte with a pH value of 4.0, the surface of PTP−AgNP/PGE may carry some positive charges, which is beneficial for the enrichment of negatively charged nitrite through electrostatic attraction. Furthermore, the possible hydrogen bond between the PTP and nitrite may be another catalyst for the enrichment of nitrite on the sensing interface. The co-function of AgNPs and PTP causes the oxidation peak current of NO_2_^−^ to increase greatly.

#### 3.4.2. Electrocatalysis Mechanisms of PTP-AgNP Film

**Figure 6:**
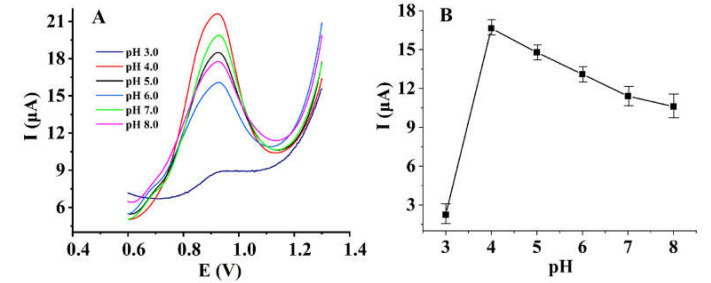
Differential pulse voltammograms of 1 mM nitrite on PTP−AgNP/PGEs in 0.1 M PB with differing pH values (**A**), and the influence of the pH value of the PB solution on the oxidation peak current of nitrite (**B**).

The pH value of the supporting electrolyte not only affects the interface state of the sensor, but also the dissociation state of the target. Therefore, it has a high influence on the electrochemical reaction mechanism of the target. Through using differential pulse voltammetry, the influence of the pH value of the 0.1 M PB solution on the electrochemical oxidation of nitrite was studied, and the results are shown in Figure 6A. Evidently, the oxidation peak potential of nitrite changes little alongside the change in pH value, which indicates that the reaction is a process without proton participation. Moreover, compared with other pH values, when the pH value of the supporting electrolyte is 4.0, the sensor shows a higher sensitivity for the detection of nitrite (Figure 6B). Presumably, in this environment, the surface of the sensor is in a better state for the enrichment of nitrite. Therefore, a 0.1 M PB solution with a pH value of 4.0 was chosen as the supporting electrolyte.

**Figure 7:**
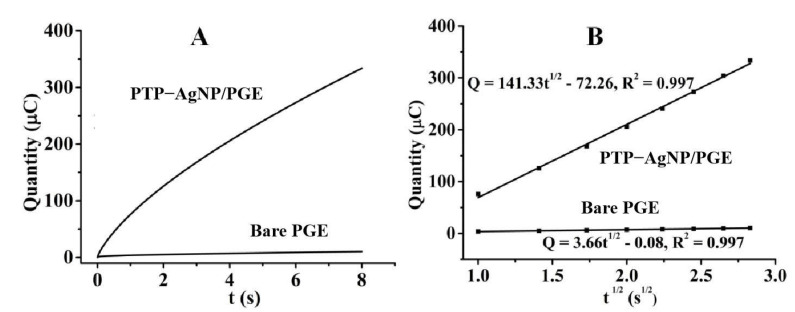
The relationship between Q and t for bare PGE and PTP−AgNP/PGE (**A**) and the linear relationship between Q and t^1/2^ for bare PGE and PTP−AgNP/PGE (**B**).

The influence of PTP–AgNP nanocomposite film on the adsorption quantity of nitrite on the electrode surface was studied with chronocoulometry; the results are shown in Figure 7A. The Cottrell equation, as shown in Equation (1), describes the relationship between Q and t^1/2^ well [39].

(1) $${\;Q=2\mathrm{ncFAD}}_{0}^{1/2}t^{1/2}\pi^{-1/2}+Q_{\mathrm{dl}}+\mathrm{nFA}\Gamma_{0}$$

In Equation (1), n refers to the number of transfer electrons, c is the concentration of the target, F is the Faraday constant (96500 C/mol), A is the electrode area (cm^2^), D is the diffusion coefficient (cm^2^/s) of target, Q_dl_ is the charging capacity of the electric double layer (C), nFAΓ_0_ is the Faraday component (C), and Γ_0_ is the surface coverage. Combining Equation (1) with the data analysis results shown in Figure 7B, the amount of nitrite on PTP−AgNP/PGE is calculated to be about 39 times that of bare PGE, which indicates the good sensitivity of the sensor.

**Figure 8:**
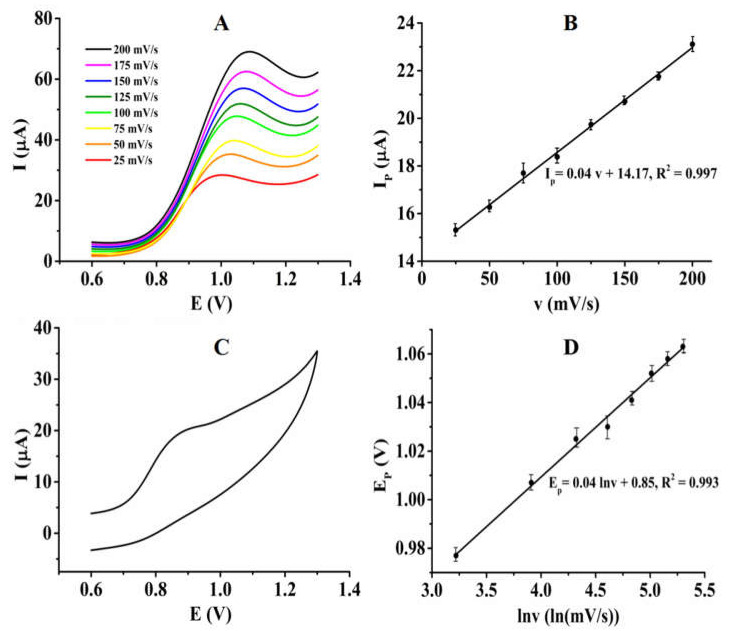
(**A**) Linear sweep voltammograms of PTP–AgNP/PGE in 0.1 M phosphate buffer solution (pH = 4.0) containing 2 mM NO_2_^−^ at different scan rates. (**B**) Linear relationship of the oxidation peak currents of NO_2_^−^ (I_p_) with scan rates. (**C**) Cyclic voltammogram of 2 mM NO_2_^−^ on PTP–AgNP/PGE in 0.1 M PB (pH = 4.0). (**D**) Linear relationship of the oxidation peak potentials of NO_2_^−^ (E_p_) with the natural logarithm of scan rates.

Linear sweep voltammetry was used to study the electrochemical-reaction kinetic process of nitrite on the PTP−AgNP/PGE. As shown in Figure 8A, the oxidation peak currents of NO_2_^−^ increased linearly with scan rates in the range of 25–200 mV/s (Figure 8B), which indicates that the electrode reaction is controlled by adsorption. Moreover, this was also found to be an irreversible process (Figure 8C). According to Laviron’s theory [40], for an adsorption-controlled and complete irreversible electrochemical reaction, Equation (2) describes the relationship between the peak potential and the natural logarithm of scan rate well:

(2) $$E_{p}=E^{0\prime }+\frac{\mathrm{RT}}{\alpha nF}\ln\frac{\mathrm{RT}k^{0}}{\alpha nF}+\frac{\mathrm{RT}}{\alpha nF}\ln v$$

where E^0′^ is the formal potential, α is the electron transfer coefficient, k^0^ is the standard rate constant, n is the electron transfer number, and F is the Faraday constant. For completely irreversible adsorption-controlled processes, α is generally taken as 0.5. In this system, the relationship between the peak potential and the natural logarithm of the scan rate is E_p_ (V) = 0.04 lnv (mV/s) + 0.85, R^2^ = 0.993 (Figure 8D). According to Equation (2), the number of electrons transferred can be calculated as n = 1.28 ≈ 1. Therefore, the electrochemical oxidation reaction of nitrite on PTP−AgNP/PGE is an adsorption controlled process that transfers about one electron. The electrochemical-reaction mechanism of nitrite on the sensing interface was hypothesized as follows:

NO_2_^−^ → NO_2_ + e

#### 3.4.3. Performances of the Nitrite Sensor

**Figure 9:**
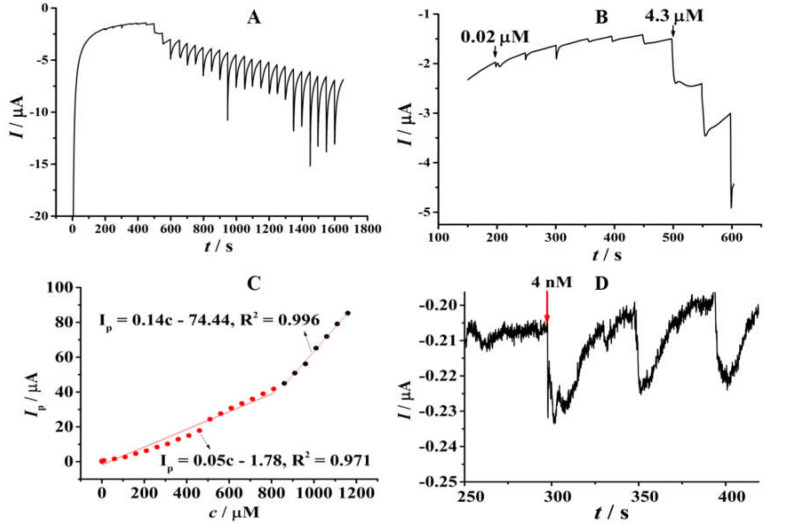
(**A**) Amperometric responses of different concentrations of nitrite in PTP−AgNP/PGE in a 0.1 M phosphate buffer (pH = 4.0). (**B**) Amperometric responses in a lower concentration range. (**C**) The linear relationship between the oxidation peak currents of nitrite and its concentrations. (**D**) Detection limit of the sensor. Applied potential: 0.9 V.

The linear range, response time, sensitivity and detection limit of the sensor were evaluated in a 0.1 M phosphate buffer (pH = 4.0) through the amperometric I-t curve method. Figure 9A shows the experimental results and Figure 9B shows the amperometric response in a lower concentration range. As shown in Figure 9C, the linear range of this nitrite sensor can be divided into two segments. One is in a lower concentration from 0.02 μM to 860 μM with a linear equation of I_p_ (μA) = 0.05 c − 1.78, R^2^ = 0.971, indicating a sensitivity of 50 nA/μM. The other is in a higher concentration from 860 μM to 1160 μM, I_p_ (μA) = 0.14 c − 74.44, R^2^ = 0.996, indicating a sensitivity of 140 nA/μM. Moreover, the detection limit and response time of the sensor were measured to be 4 nM and 2 s in the higher concentration, respectively (Figure 9D). Compared with other reported nitrite electrochemical sensors (Table 1), the sensor fabricated in this work shows obvious advantages, including a wider linear range, higher sensitivity, and a lower detection limit.

With the aid of differential pulse voltammetry, the stability and reproducibility of the sensor were investigated. After placing the sensor in open air for 7 days, a parallel measurement was performed. Results showed that the electrochemical signal could still maintain 94% of its initial signal, indicating that the sensor has good stability (Figure S1). In applying the same sensor to detect 2 mM nitrite 6 times, the relative standard deviation of the determination results was calculated to be 1.4%. Moreover, in detecting 2 mM nitrite with five different sensors, the relative standard deviation of the detection results was calculated to be 8.3%, which indicates that the sensor has good reproducibility (Figure S2).

**Figure 10:**
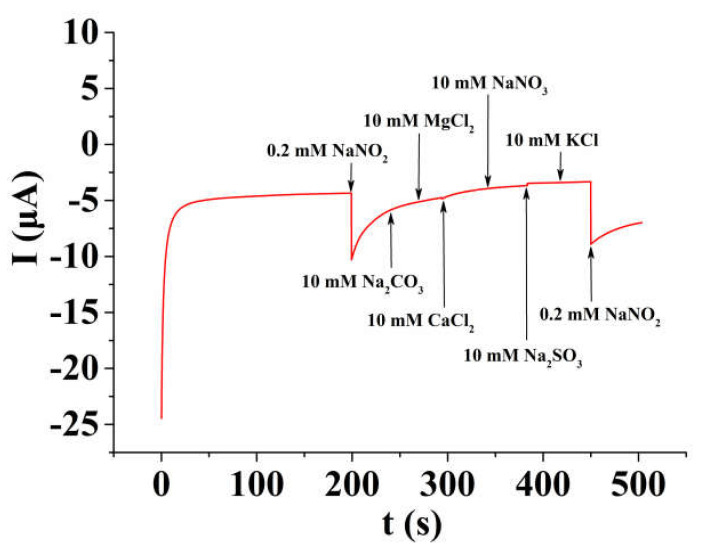
Amperometric responses of 0.2 mM nitrite and other possible coexisting interferents on PTP−AgNP/PGE in 0.1 M PB (pH = 4.0) with the operation potential of 0.9 V.

In order to ensure the accuracy of the sensor, its selectivity was assessed through the amperometric I-t curve method. With continuous stirring during the experiment, a potential total of 0.9 V, 0.2 mM NaNO_2_, 10 mM NaCO_3_, 10 mM MgCl_2_, 10 mM CaCl_2_, 10 mM NaNO_3_, 10 mM Na_2_SO_3_, 10 mM KCl, and 0.2 mM NaNO_2_ was added to 5 mL of 0.1 M PB solution (pH = 4.0) about every 40 s. As shown in Figure 10, 50 folds of CO_3_^2−^, NO_3_^−^, SO_3_^2−^, K^+^, Ca^2+^, and Mg^2+^, and 100 folds of Na^+^ and Cl^−^ have no interference on the detection of 0.2 mM NO_2_^−^, indicating the good selectivity of the sensor.

#### 3.4.4. Measurement of Nitrite in Food Sample

To evaluate its practical application ability, the sensor was applied for the determination of nitrite in food samples. The food samples were purchased from a local supermarket (Wuhan, China) and processed by the Wuhan Institute for Food and Cosmetic Control following the second method of GB 5009.33-2016. Each sample was measured with the sensor in parallel five times and the determination results were compared with the results from spectrophotometry. As shown in Table 2, the nitrite sensor has good detection accuracy and may have good application prospects.

## 4. Conclusions

In this work, AgNPs were synthesized via tea extract and modified on the surface of PGEs through co-electropolymerization with tea polyphenols. This method is not only environmentally friendly, but also simple and controllable. The fabricated PTP–AgNP nanocomposite film has clear electrocatalytic effects on the oxidation of nitrite, and the modified PGE can work as a disposable amperometric nitrite sensor. Electrochemical techniques, spectral technology, and SEM and TEM technology were applied to perform the characterizations, study the sensing mechanisms, and evaluate the performance of the sensor. The sensor boasts a wide linear range (0.02–1160 μM), a low detection limit (4 nM), a short response time (2 s), and good selectivity, stability, and reproducibility. Furthermore, it can be applied toward the accurate and timely detection of nitrite in food samples, which indicates its good application prospects.

## Figures and Tables

**Table 1:** Comparison of the performances of different electrochemical sensors for nitrite.

Electrode	Linear Range (μM)	Sensitivity (μA/μM)	Detection Limit (μM)	Reference
Facile synthesis of graphene oxide–silver nanocomposite-modified glassy carbon electrode	10–180	/	2.1	[41]
Cytochrome c-type nitrite redutase/Nafion/methylviologen-modified glassy carbon electrode	75–800	/	60	[42]
Silver particle–ionic liquid-modified carbon paste electrode	50–1000	/	3	[43]
Reduced graphene/Pd nanocomposite-modified glassy carbon electrode	0.536–108	0.06	0.015	[44]
Nafion/single-layer graphene nanoplatelet-tetrasodium 1,3,6,8-pyrenetetrasulfonic acid-myoglobin-modified glassy carbon electrode	50–2500	0.0006	10	[45]
Reduced graphene oxide-modified screen-printed electrode	20–100 100–1000	0.015 0.007	0.83	[46]
Platinum nanoparticles/4-benzenamine (2-aminoethyl)/3-mercaptopropionic acid-modified gold electrode	10–1000	0.02	5	[47]
Cobalt nanoparticles/single-walled carbon nanotube-modified edge plane pyrolytic graphite electrode	32–200	0.25	5.61	[48]
Poly-(melamine) modified glassy carbon electrode	10–400	0.01	1.86	[49]
Graphene oxide/poly (3,4-ethylenedioxythiophene) poly-(styrenesulfonate) modified glassy carbon electrode	1–50 50–200	0.026 0.016	0.5	[50]
Polyaniline-linked tetra amino cobalt (II) phthalocyanine surface-functionalized ZnO hybrid nanomaterial-modified glassy carbon electrode	50–500	0.45	0.021	[51]
Metal-organic gel-multiwalled carbon nanotube nanocomposite-modified glassy carbon electrode	0.3–100	0.037	0.086	[52]
Gold-Tungsten bimetallic nanoparticle-decorated graphene-chitosan-modified pencil graphite electrode	10–250	0.9	0.12	[53]
Chloroperoxidase-ionic liquid/reduced graphene oxide-gold nanoparticle-modified glassy carbon electrode	0.5–300	0.05	0.049	[54]
Poly-(tea polyphenols) silver nanoparticle-modified pencil graphite electrode	0.02–860 860–1160	0.05 0.14	0.004	This work

**Table 2:** Determination of nitrite in food samples.

Samples	Nitrite Found (μM ± RSD, n = 5)	
	The Present Method	Spectrophotometry
Duck feet	0.183 ± 0.07	0.178 ± 0.06
Sausage	2.263 ± 0.23	2.257 ± 0.31
Bacon	0.257 ± 0.05	0.250 ± 0.04

## Data Availability

Not applicable.

## Supplementary Materials

The following supporting information can be downloaded at: https://www.mdpi.com/article/10.3390/mi14030687/s1, Figure S1: Differential pulse voltammograms of 1 mM nitrite on PTP-AgNPs/PGE in 0.1 M phosphate buffer (pH = 4.0) at different time, Figure S2: Parallel determination of 1 mM nitrite with one PTP-AgNPs/PGE in 0.1 M phosphate buffer (pH = 4.0) for six times (A) and parallel determination of 1 mM nitrite with five PTP-AgNPs/PGEs in 0.1 M phosphate buffer (pH = 4.0) (B).
