# Supplementary material for: Fabrication of a Disposable Amperometric Sensor for the Determination of Nitrite in Food
Source: Micromachines (Basel). 2023 Mar 20;14(3):687. doi: 10.3390/mi14030687 (PMC10056392; doi:10.3390/mi14030687)
Supplement: Supplementary file 1 [file micromachines-14-00687-s001.zip › micromachines-2290003-supplementary.pdf]

Supporting Information for

Fabrication of a Disposable Amperometric Sensor for the Determination of Nitrite in Food

Chao Liu, Daoming Chen, Chunnan Zhu, Xiaojun Liu, Yu Wang, Yuepeng Lu, Dongyun Zheng*, Baorong Fu*

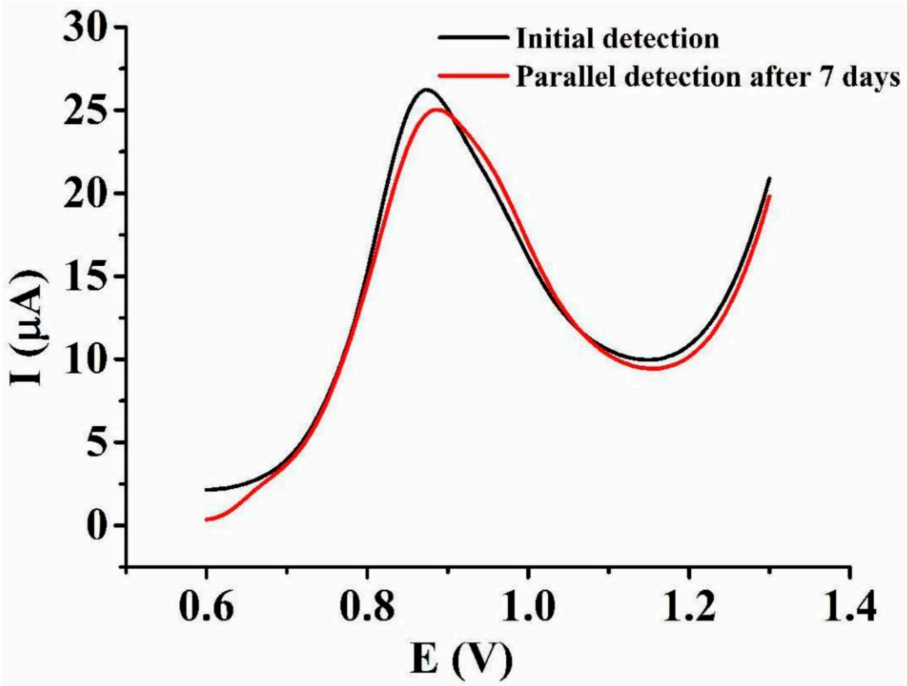

Figure S1. Differential pulse voltammograms of 1 mM nitrite on PTP-AgNP/PGE in 0.1 M phosphate buffer (pH = 4.0) at different time.

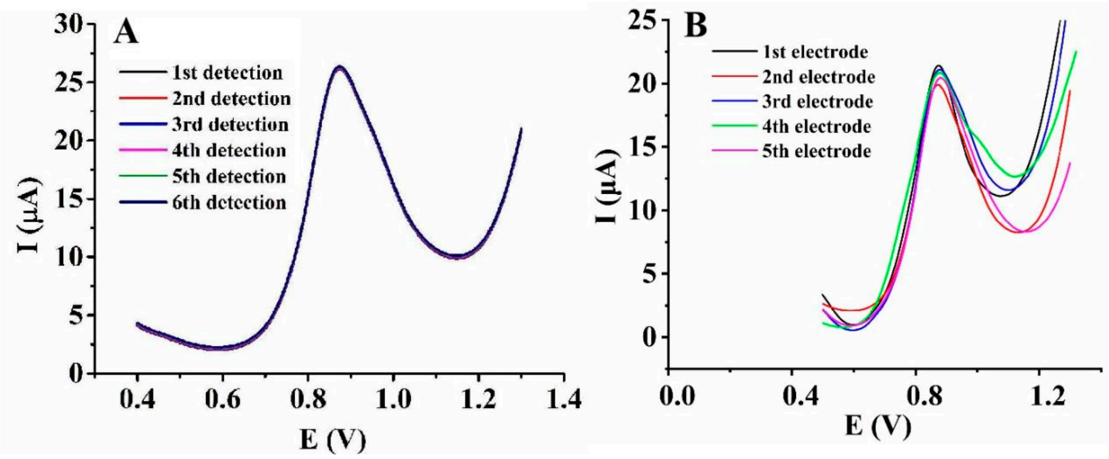

Figure S2. Parallel determination of 1 mM nitrite with one PTP-AgNP/PGE in 0.1 M phosphate buffer (pH = 4.0) for six times (A), and parallel determination of 1 mM nitrite with five PTP-AgNP/PGEs in 0.1 M phosphate buffer (pH = 4.0) (B).
